# Direct Reprogramming—The Future of Cardiac Regeneration?

**DOI:** 10.3390/ijms160817368

**Published:** 2015-07-29

**Authors:** Stefanie A. Doppler, Marcus-André Deutsch, Rüdiger Lange, Markus Krane

**Affiliations:** 1 Division of Experimental Surgery, Department of Cardiovascular Surgery, Deutsches Herzzentrum München, Technische Universität München (TUM), Munich 80636, Germany; E-Mails: deutsch@dhm.mhn.de (M.-A.D.); lange@dhm.mhn.de (R.L.); krane@dhm.mhn.de (M.K.); 2 DZHK (German Center for Cardiovascular Research)—Partner Site Munich Heart Alliance, Munich 80802, Germany

**Keywords:** direct reprogramming, transdifferentiation, Gata4, Mef2c, Tbx5 (GMT), induced cardiomyocytes (iCMs)

## Abstract

Today, the only available curative therapy for end stage congestive heart failure (CHF) is heart transplantation. This therapeutic option is strongly limited by declining numbers of available donor hearts and by restricted long-term performance of the transplanted graft. The disastrous prognosis for CHF with its restricted therapeutic options has led scientists to develop different concepts of alternative regenerative treatment strategies including stem cell transplantation or stimulating cell proliferation of different cardiac cell types *in situ*. However, first clinical trials with overall inconsistent results were not encouraging, particularly in terms of functional outcome. Among other approaches, very promising ongoing pre-clinical research focuses on direct lineage conversion of scar fibroblasts into functional myocardium, termed “direct reprogramming” or “transdifferentiation.” This review seeks to summarize strategies for direct cardiac reprogramming including the application of different sets of transcription factors, microRNAs, and small molecules for an efficient generation of cardiomyogenic cells for regenerative purposes.

## 1. Introduction

Since cardiomyocytes have a very limited capacity to divide in the adult heart, wound healing after cardiac injury results in fibrotic scar formation at the affected site rather than cardiac muscle regeneration [1,2,3]. Pharmacological, interventional, and operative therapies are not able to compensate for the irreversible and massive loss of cardiomyocytes associated with myocardial infarction or other cardiac diseases [4]. Still, the only true cure for heart failure is whole organ transplantation, but this opportunity is extremely limited by the number of donor hearts and further issues like immunosuppressant therapy or graft vasculopathy [1,5]. Current clinical trials addressing stem cell-based therapies have failed to reach expectations, especially when looking at functional improvements [4]. Thus, novel and “true” cardiac regenerative therapies are in high demand. Cardiac transplantation of different cell types combined with tissue engineering, a stimulation of endogenous pro-regenerative mechanisms, like forcing resident cardiomyocytes to re-enter the cell-cycle by cytokine delivery or *in situ* direct reprogramming of cardiac cells, are conceivable concepts.

The generation of pluripotent cells from different adult somatic cell types, referred to as reprogramming and developed by Takahashi and Yamanaka (2006) [6], is nowadays performed routinely in many laboratories all over the world. For this outstanding concept Shin’ya Yamanaka received the Nobel Prize in Physiology or Medicine in 2012, together with John Gurdon [7]. Triggered by the advent of this technology, a few years later the idea of a direct conversion from one determined cell type into another, without going through a pluripotent stage, simply by overexpressing transcription factors or microRNAs, reemerged. As early as 1987, Davis and colleagues [8] already induced myogenic features in fibroblasts by ectopic expression of the muscle-specific transcription factor *MyoD*. This direct conversion process is characterized by a progressive activation of the target cell program while concomitantly suppressing the starting cell profile [9]. Meanwhile, successful direct reprogramming has been reported for many cell types like pancreatic beta cells [10], neurons [11], hepatocyte-like cells [12,13], or hematopoietic progenitor cells [14]. However, the ambitious aim is to generate functional cardiomyocytes directly from fibroblasts. Ieda *et al.* [15] were the first who reported successful direct transdifferentiation of murine fibroblasts into functional cardiomyocytes, also termed induced cardiomyocytes (iCMs) in 2010. However, cardiomyocytes are a very complex cell type with elaborate sarcomeric structures. In their mature form they usually do not divide and *in situ* they are integrated in an advanced electrophysiological network. These are only some of the issues that have to be addressed when trying to generate functional iCMs.

This article seeks to comprehensively review different strategies for direct cardiac reprogramming by not only elucidating the possibilities for cardiac regeneration but also discussing the remaining challenges before a clinical application may become reality.

## 2. Direct Lineage Reprogramming/Conversion of Fibroblasts into Cardiomyocytes *in Vitro*

### 2.1. The Starting Cell Population—Why Fibroblasts?

Apart from cardiomyocytes, the adult heart consists of many noncardiogenic cell types, the majority of which are fibroblasts [2]. However, amounts of cardiac fibroblasts can be extremely different across species. In the adult mouse heart about 30% of the cardiac cells contribute to the fibroblastic cell mass, whereas in the adult rat heart more than 60% of all cells are fibroblasts [16]. Banerjee *et al.* explain these tremendous differences by Laplace’s law. Since the rat heart has a bigger ventricular wall radius, it is subjected to more tension caused by blood pressure and thus needs more connective tissue (built up by fibroblasts) to stabilize the ventricular wall.

In the healthy heart cardiac fibroblasts already play a major role for structural and paracrine support of their adjacent myocytes [17]. However, after myocardial injury, resident fibroblasts are activated and migrate to the site of injury, where they create scar tissue in order to maintain the structural integrity of the heart but unfortunately without contractile ability [2,3]. The abundance of cardiac fibroblasts in the injured heart predestines them as a target for reprogramming approaches, implying *in situ* regeneration of the myocardium [2]. Another important reason for cardiac fibroblasts to serve as target cells for a direct conversion into cardiomyocytes is the fact that both cell types derive from a common progenitor cell population and thus likely share some epigenetic features [1,18]. The importance of the originating cell type and their native environment was, for example, reported for myogenic or pancreatic β-cell reprogramming. *MyoD* (myogenic differentiation 1) is a transcription factor that can directly convert fibroblasts into skeletal myocytes. However, when *MyoD* was overexpressed in retinal pigment epithelial cells, melanocytes, or hepatocytes, all of which originate from different germ layers, skeletal muscle reprogramming failed [19]. The same holds true for pancreatic β-cell reprogramming. *Ngn3*, *Pdx1*, and *Mafa* were indeed able to efficiently reprogram pancreatic exocrine cells into functional β-cells *in vivo*, but the same factors were insufficient to convert fibroblasts *in vitro* [10]. For cardiac reprogramming approaches, a wide range of fibroblastic cell types, like murine embryonic fibroblasts, tail-tip fibroblasts, cardiac fibroblasts, human foreskin fibroblasts, or dermal fibroblasts have been used with variable success (see Table 1 and Table 2). The choice of one of these, quite heterogenic, fibroblast populations may affect direct reprogramming by the fibroblasts’ specific properties or their isolation protocols. Using cardiac fibroblasts as a starting population entails the risk of contaminating cardiomyocytes or cardiac progenitor cells since neither a truly specific marker nor a method for truly purifying cardiac fibroblasts exists [20]. Contaminating cardiomyocytes or cardiac progenitor cells could therefore be the cause of rare beating events observed in direct cardiac reprogramming approaches. The same holds true for embryonic fibroblasts, which are immature cells and by that may still contain a rather high plasticity potential but could also be contaminated by cardiac progenitor cells. By using tail-tip or dermal fibroblasts for direct reprogramming, contamination problems could be ruled out, but unfortunately most researchers were not able to induce beating events in such fibroblasts (e.g., [15,21], see also Table 1 and Table 2). However, residual epigenetic memory of cardiac fibroblasts could further contribute to the fact that this initial cell population is easier to convert to iCMs than other fibroblast populations. Sekiya *et al.* [13], for example, demonstrated that during the conversion of murine fibroblasts to hepatocytes driven by *Hnf4α* and *Foxa1*, fibroblast identity was not completely silenced.

**Table 1:** Direct cardiac reprogramming *in vitro* (mouse).

Reference	Cell Source	Reprogramming Factors	Markers of iCMs, Percentages		Spontaneous Beating/When	Comments
[15]	**MCFs**, **TTFs**	Starting with 14 F: GMT best	**αMHC-GFP + TropT** (FACS, day 7)		**MCFs**: yes, after 4–5 weeks (rare events)	No CPC stage
			**MCFs**:	GMT: ~4%–6%		
			**TTFs**:	GMT: ~2.5%		
[22]	**aMCFs**, **aTTFs**	H_2_GMT	**αMHC-GFP + TropT** (FACS)		**aMCFs**: yes, after 5 weeks (rare events) **aTTFs**: yes, after 5 weeks (rare events)	–
			**aTTFs** (day 9):	GMT: ~2.9%		
				H_2_GMT: ~9.2%		
			**aMCFs** (day 7):	GMT: ~1.4%		
				H_2_GMT: ~6.8%		
[23]	**aMCFs**, **aTTFs**	GMT	**αMHC-GFP**; **Nkx2.5-GFP** (FACS, day 21): no GFP+ cells		No beating	no CPC stage (Nkx2.5-GFP)
[21]	**MEFs** (E13.5, w/o head, w/o visceral organs, p3–p5) **MCFs** (aMHC-tdTomato mice: Thy1^+^, tdTomato^-^) **TTFs** (3-day-old mice)	MTMc	**MEFs**: for initial qRT-PCR screening		**MCFs**: no, after 4 weeks (only rare events which were considered as cardiomyocyte contamination)	–
			**αMHC-tdTomato** (FACS, day 14)			
			**MCFs**:	GMT: 2.2% ± 0.2%		
				MTMc: 2.5% ± 0.3%		
			**TTFs**:	GMT: 2.4% ± 0.2%		
			**TropT** (FACS, day 14)			
			**MCFs**:	GMT: 12% ± 3.7%		
				MTMc: 11% ± 2.0%		
[24]	**MCFs**	miR-1, miR-133, miR-208, miR-499 + JI1	**αMHC-CFP** (FACS, day 7)		**MCFs**: yes, after 10 days (rare events 1%–2% of total cell population)	short CPC stage: Mesp2 from day 1–5 (miR-1, -133, -208, -499); no pluripotency marker detected (Oct4, Nanog)
			negmiR: 0.1%–3.9%			
			miR-1, -133, -208, -499: 1.1%–5.3%			
			negmiR + JI1: 0.3%–1.7%			
			miR-1, -133, -208, -499 + JI1: 13.4%–27.9%			
[25]	**MEFs** (E14.5, w/o head, w/o visceral organs, w/o heart, p3–p5) **aMCFs** (8–10-week-old mice), isolation by culture, p3	NH_2_GMT	**TropT-GCaMP** (Ca^2+^ oscillation, day 14)		**MEFs**: yes, after day 14	iCMs not proliferative (Ki67)
			**MEFs**:	GMT: 0.03% ± 0.02%		
				NH_2_GMT: 1.6% ± 0.3%		
			**MCFs**:	NH_2_GMT: 4.5% ± 0.3%		
[26]	**MEFs** (E13)	MpScMcSfNH_1_H_2_GTM	**αMHC-GFP** (FACS, day 7)		N.A.	–
			NegCtr: 0.03% ± 0.05%			
			GMT: 0.05% ± 0.06%			
			GMTMcSf: 1.60% ± 0.12%			
			GMTMpSc: 0.20% ± 0.07%			
			GMTMcSfMpSc: 2.40% ± 0.11%			
[27]	**MEFs** (E14.5, w/o head, w/o visceral organs, w/o heart), p3–p5 **aMCFs** (8–10-week-old mice), isolation by culture, p3	NH_2_GMT + small molecules (SB)	**TropT-GCaMP** (Ca^2+^ oscillation, day 14)		**MEFs**: robust beating, after day 11 **aMCFs**: yes, after day 16	TGFβ signaling pathway plays a role in conversion
			**MEFs**:	NH_2_GMT + DMSO: 5.0% ± 1.8%		
				NH_2_GMT + SB: 17.0% ± 0.4%		
			**aMCFs**:	NH_2_GMT + DMSO: 1.5% ± 0.4%		
				NH_2_GMT + SB (5 µM): 9.3% ± 1.3%		
[9]	**MEFs** (E12.5, w/o head, w/o visceral organs) **aMCFs** (adult αMHC-GFP mice; Thy1^+^ GFP^−^)	GMT + miR133	**αMHC-GFP** (FACS, day 7)		**MEFs**: GMT: yes, after 4 weeks; GMT + miR133: yes, after 10 days **aMCFs**: N.A.	iCMs not proliferative (EdU assay) Snai1/EMT mechanism no Mesp1+ CPCs (Mesp1-Cre x Stop-GFP mouse MEFs) mainly atrial-type myocytes
			**MEFs**:	GMT: ~19%; GMT + miR133: ~33%		
			**TropT** (FACS, day 7)			
			**MEFs**:	GMT: ~1.9%; GMT + miR133: ~12%		
[28]	**MEFs**, **TTFs**, **MCFs**	20 F H_2_GMT	**Hcn4-GFP** (FACS, day 7)		No (due to inadequate sarcomeric protein expression and organization, 12 weeks of culture) 0.0%–0.16% of H_2_GMT transduced fibroblasts show spontaneous beating (no further specification)	No Nkx2.5+ CPCs; well organized sarcomeric structures necessary for spontaneous beating, H_2_GMT: different types of CMs (atrial, pacemaker, and ventricular)
			**TTFs**:	20F: 15%;		
				G_6_T_3_TR: ~40%		
				H_2_GMT: N.A.		
[29]	**MCFs** (1.5 day old mice), **TTFs**	GMT (polycistronic vector, different order)	**αMHC-GFP or TropT** (FACS, day 10)		**MCFs**: MGT: yes, after 3 weeks	Stoichiometry is of critical importance (especially high Mef2c levels)
			**MCFs**:	G + M + T: ~5% (GFP), ~0.2% (TropT)		
				GMT: ~1% (GFP), 0.02% (TropT)		
				MGT: ~10% (GFP), ~4.8% (TropT)		
				MTG: ~9% (GFP), ~3.5% (TropT)		
[30] **CASD approach**	**MEFs**, **TTFs**	OSKMy + cytokines + small molecules	**Nebulette-LacZ**: initial screening		**MEFs**: yes, after day 11 **TTFs**: yes, after day 12 beating patches generated per 100,000 cells on day 18 **MEFs**: 145 ± 6 **TTFs**: 115 ± 7	CPC stage: on day 9–10 (Flk1, Nkx2.5, Gata4) day 11: only atrial CMs only Mlc2a not Mlc2v
			**MEFs**: OSK + JI1 (day 1–day 9) + BMP4 (day 9–day 14): N.A.			
			**TropT** (FACS, day 18)			
			**TTFs**: OSKM + JI1 (day1–day9) + BMP4 (day 9–day 14), 39% ± 2%			
[31] **CASD approach**	**MEFs** (E13.5, w/o head, w/o visceral organs, w/o heart), **TTFs**	Oct4 + small molecules (SCPF) + BMP4	**Beating cluster** (day 30)		**MEFs**: yes, after day 20 **TTFs**: yes	CPC stage mostly ventricular iCMs (Mlc2v)
			**MEFs**: 99 ± 17 per 10,000 starting cells			
			**TTFs**: ~50 per 10,000 starting cells			

Abbreviations: iCMs, induced cardiomyocytes; CASD, Cell-Activation and Signaling-Directed; Cell Source, MEFs: murine embryonic fibroblasts; MCFs, murine cardiac fibroblasts; TTFs, tail-tip fibroblasts; a, adult; w/o, without; p, passage; Reprogramming Factors, F: factors; O, Oct4; S, Sox2; K, Klf4; My, c-Myc; H_2_, Hand2; N, Nkx2.5; G, Gata4; G_6_, Gata6; M, Mef2c; T, Tbx5; T_3_, Tbx3; Mc, Myocardin; Mp, Mesp1; Sc, Smarcd3 (Baf60c); Sf, SRF; H_1_, Hand1; E, ESRRG; Z, ZFPM2; R, Rxra; JI1, JAK inhibitor JI1; BMP4, bone morphogenic protein 4; SB, SB431542 (TGFβ-inhibitor); S, SB431542 (ALK4/5/7 inhibitor); C, CHIR99021 (GSK3 inhibitor); P, parnate (LSD1/KDM1 inhibitor); F, forskolin (adenylyl cyclase activator); Comments: CPC, cardiac progenitor cell. N.A., not available; bold text, markers for iCMs and used cell sources.

**Table 2:** Direct cardiac reprogramming *in vitro* (human).

Reference	Cell Source	Reprogramming Factors	Markers of iCMs, Percentages			Spontaneous Beating/When	Comments
[32]	**HFFs**, **aHCFs**, **aHDFs**	GMTH_2_Mc + miR-1, miR-133	**TropT** (FACS, day 14)			Yes (only from **HCFs**), after 11 weeks	–
			**HFFs**:		GMT: failed		
					GMTH_2_: failed		
					GTMcH_2_ + miR-1/133: 20%		
			**aHCFs**:		GTMcH_2_ + miR-1/133: 13%		
			**aHDFs**:		GTMcH_2_ + miR-1/133: 9.5%		
			**Ca^2+^ transients** (4 weeks)				
			**aHCFs**:		~15%		
[33]	**HCFs** (THY1^+^, CD31^−^) **HFFs**	GMTMpMc	**TropT, αActinin** (FACS, 4 weeks)			GMTMpMc-**HCFs**: no beating during longer periods of culture (no exact time designation) GMTMpMc-**HCFs** + ratCMs co-culture: beating after 7 days GMTMpMc-**HCFs** + conditioned medium: no beating	–
			**HCFs**:		GMT: not sufficient		
					GMTMpMc: ~5%		
			**Ca^2+^ oscillation** (4 weeks)				
			**HCFs**:		GMTMpMc: ~1%		
[34]	**H9Fs** (THY1^+^), **HDFs**, **HCFs**	7F (GMTMpMcEZ) 5F (GMTMpE)	**αMHC-mCherry** (FACS, day 14)			No beating events reported	**H9Fs**: well-organized sarcomeres after 10 weeks
			**H9Fs**:		7F (GMTMpMcEZ): 18.1% ± 11.2%		
			**H9Fs**:		5F (GMTMpE): lower than for 7F		
			**αMHC-mCherry + TropT** (day 14) (FACS)				
			**H9Fs**:		7F (GMTMpMcEZ): 13.0% ± 9.3%		
			**HDFs/HCFs**:		7F (GMTMpMcEZ): 1%–4%		
[9]	**HCFs** (p1–p3)	GMTMpMc, miR-133	**TropT**, **αActinin** (FACS, day 7)			N.A.	–
			**HCFs**:	GMTMpMc: 2%–8%			
				GMTMpMc + miR-133: 23%–27%			

Abbreviations: iCMs, induced cardiomyocytes; Cell Source: HFFs, human foreskin fibroblasts; HDFs, human dermal fibroblasts; HCFs, human cardiac fibroblasts; aHDFs, adult human dermal fibroblasts; aHCFs, adult human cardiac fibroblasts; H9Fs, 42 days differentiated human fibroblasts from the H9 ES cell line; p, passage; Reprogramming factors, F, Factor; G, Gata4; M, Mef2c; T, Tbx5; H_2_, Hand2; Mc, Myocardin; Mp, Mesp1; E, ESRRG; Z, ZFPM2; miR, microRNA; bold text, markers for iCMs and used cell sources; N.A., not available.

### 2.2. Reprogramming Factors—Transcription Factors and/or MicroRNA

**Figure 1:**
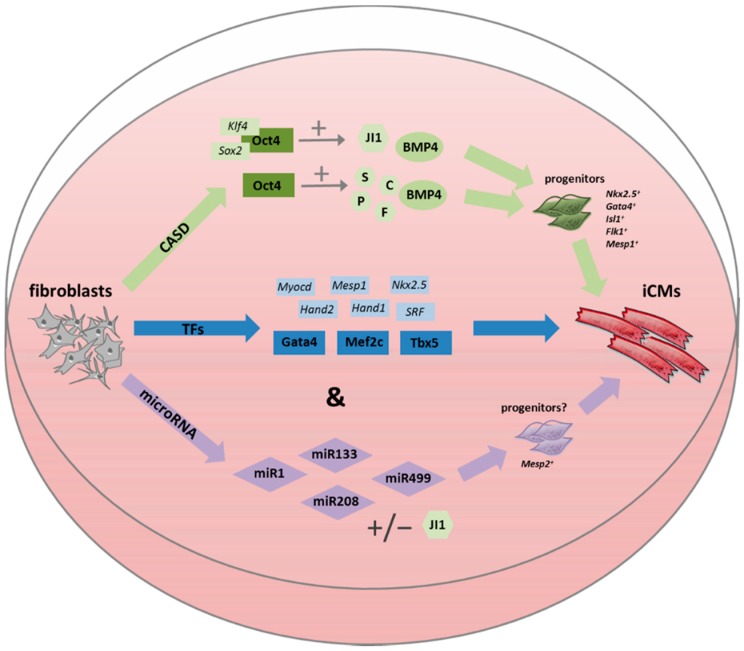
*In vitro* approaches for direct reprogramming of fibroblasts into induced cardiomyocytes (iCMs). The CASD lineage conversion method tries to directly convert fibroblasts into iCMs by a transient overexpression of pluripotency factors in combination with lineage specific soluble signals. Other, more direct approaches use transcription factors (TFs), microRNAs, or a combination of both (&) to achieve iCMs. Abbreviations: CASD: Cell-activation and signaling-directed; TF: transcription factor; miR: microRNA; iCM: induced cardiomyocyte; JI1: JAK inhibitor JI1; BMP4: bone morphogenic protein 4; S: SB431542 (ALK4/5/7 inhibitor); C: CHIR99021 (GSK3 inhibitor); P: parnate (LSD1/KDM1 inhibitor); F: forskolin (adenylyl cyclase activator).

The conventional paradigm for reprogramming or transdifferentiation is the use of target cell-type-specific transcription factors or microRNAs [31]. The first set of transcription factors reported to induce direct lineage reprogramming of fibroblasts into cardiomyocytes was *Gata4*, *Mef2c*, and *Tbx5*, well known as GMT [15]. In the following years, dozens of different reprogramming cocktails were tested, most of them based on the original combination of GMT with additional factors, e.g., *Mesp1*, *Hand1*, *Hand2*, *Nkx2.5*, *Myocardin* (*Myocd*), *Smarcd3*, or *SRF*, to improve reprogramming efficiency [22,25,26,28] (for an overview see Table 1 and Figure 1). In 2012 Chen *et al.* [23] were the first who reported that GMT alone is inefficient to produce functional cardiomyocytes but rather resulted in a partially reprogrammed cell type showing, for example, induced *TroponinT* (*TropT*) but no *αMyosin Heavy Chain* (*αMHC*) expression. Protze *et al.* (2012) [21] identified a reprogramming cocktail applying *Tbx5* and *Mef2c* in combination with *Myocd* by which ~2.5% of neonatal cardiac fibroblasts turned out to be positive for *αMHC* 14 days post transduction (GMT in their hands: 2.2%). Interestingly, the group was not able to generate beating cells after four weeks, supporting the hypothesis that complete transdifferentiation into a functional cardiomyocyte could not be achieved.

Recently, an improved efficiency of cardiac reprogramming *in vitro* as well as an improved quality of *in vitro* reprogrammed iCMs was documented after GMT was used in a polycistronic vector instead of a mixture of separate transducing viruses [29]. The authors demonstrated that the optimal balance of the three factors is of critical importance. MGT (in the form of *Mef2c*-P2A-*Gata4*-T2A-*Tbx5*, where P2A and T2A are “self-cleaving” 2a peptides) worked best since the Mef2c protein level, which seems to be crucial for an induction of a cardiomyogenic program, was relatively high in fibroblasts transduced with the MGT plasmid, whereas the expression levels for *Gata4* and *Tbx5* were rather low. This sensitive equilibrium may be the reason why GMT only worked with poor efficiency in other researchers’ experiments [35]. Two other groups [36,37] also used polycistronic GMT vector constructs *in vivo*, albeit without stoichiometric improvements and thus got more or less equimolar levels of GMT. As a consequence they only observed marginal improvements compared to the transduction of a combination of the three separate vectors. Interestingly, Inagawa and colleagues [36] observed that the polycistronic GMT retrovirus induced morphologically more mature cardiomyocyte-like cells in fibrotic tissues than those generated by injecting three separate vectors. Similar reports about the importance of stoichiometry using polycistronic vectors are found in the field of reprogramming to pluripotency. Zhang *et al.* [38] reprogrammed iPSCs from human fibroblasts using a polycistronic vector containing OKSM (*Oct4*, *Klf4*, *Sox2*, *c-Myc*) or the single factors O + K + S + M, respectively. They got nearly no reprogrammed iPSC colonies by using the polycistronic vector, although they got a higher transduction efficiency with the polycistronic construct. Since they detected equimolar ratios of Oct4, Klf4, Sox2, and c-Myc protein expression for the polycistronic construct they assumed that this may be suboptimal for inducing reprogramming. Carey *et al.* [39] also reported a reduced reprogramming efficiency down to 0.0001% by applying a polycistronic OSKM plasmid to murine embryonic fibroblasts compared to the usual 0.01%–0.1%, although they detected a lentiviral transduction efficiency of 70%. Two years later, the same group [40] published that high expression of *Oct4* and *Klf4* combined with lower expression of *c-Myc* and *Sox2* was optimal to generate high-quality iPS cells.

Nam *et al.* [28] sought to reprogram murine fibroblasts (MEFs, TTFs, MCFs) directly into pacemaker cells while using an *Hcn4*-promoter-GFP transgenic cell line for event recording. By a combination of *Gata6*, *Tbx3*, *Tbx5*, and *Rxra* they got up to 40% of GFP-positive cells but they were not able to find spontaneously beating cells, probably due to inadequate sarcomeric protein expression and organization even after a culture period of 12 weeks. On the other hand, when using GMT together with *Hand2*, beating cells with diverse phenotypes (atrial, ventricular, and pacemaker) could be induced from fibroblasts of the same genetic background. These data suggest that monitoring a single reporter gene by using only a single transgenic model might lead to misinterpretations and does not necessarily result in the desired phenotype since for cardiac development as well as mature cardiomyocytes no singular key factor exists.

Jayawardena *et al.* [24] were the first who identified a cocktail of microRNAs (miR-1, -133, -208, -499) that seemed to be able to directly convert fibroblasts into iCMs. MicroRNAs (miRs) have been described to bind to the 3′-untranslated regions (UTR) of their corresponding target mRNAs and consequently suppress their translation [9]. This regulatory mechanism plays an important role in cell fate decisions. Jayawardena *et al.* [24] observed cardiac protein expression, rhythmic calcium oscillations, and rarely beating events in about 1% or 2% of the total cell population. Interestingly, they did not use viral delivery but introduced miRs by transient transfection, which is an important step toward a clinical application of this method.

Muraoka *et al.* [9] used MEFs from *α-MHC* promoter-driven eGFP transgenic mice to confirm the effect of miR-1, -133, -208, and -499 to generate iCMs. Unfortunately, they were not able to induce iCMs using this defined cocktail of miRs but combining GMT transcription factors with miR-133 resulted in significantly enhanced cardiac reprogramming efficiencies in murine and human fibroblasts. When they tested for the myocyte subtype by immunostaining in reprogrammed murine embryonic fibroblasts, they observed mostly atrial-type myocytes. Interestingly, they described beating events in GMT + miR-133 transduced MEFs as early as on day 10 post-induction, whereas GMT alone induced beating not before four weeks.

### 2.3. Cell-Activation and Signaling-Directed (CASD) Lineage Conversion Method

A different approach uses a combination of cell-activation by a transient overexpression of pluripotency factors (usually established for iPSC generation) in conjunction with lineage-specific soluble signals to directly reprogram somatic cells into diverse lineage-specific cells but without entering the pluripotent state [31]. Efe *et al.* [30] first used a *Nebulette*–LacZ reporter construct to monitor the induction of early nascent myocardium in MEFs by retroviral overexpression of *Oct4*, *Sox2*, and *Klf4* (OSK) combined with LIF-free medium conditions, 5% FCS and culture of cells on Matrigel or Geltrex. As a next step they sought to induce spontaneously contracting patches of cardiac cells and therefore treated OSK-transduced MEFs with cytokines and small molecules during the reprogramming process (chemically defined media). As their best combination the small-molecule JAK inhibitor JI1 was added to the reprogramming medium during the initial nine-day period and from day nine onwards BMP4 was supplemented (see also Table 1). The group observed an upregulation of mid-stage cardiac progenitor markers such as *Flk1*, *Nkx2.5*, and *Gata4* at day nine to 10 after transduction, followed by an expression of typical late-stage markers like *TropT*, *αMHC*, or *αActinin* from day 11. Interestingly, they only detected the atrial isoform of *Myosin light chain* (*Mlc2a*) but not the ventricular form (*Mlc2v*), regardless of the cultivation time, suggesting that they only generated iCMs of the atrial subtype by this method. This was later confirmed by electrophysiological measurements after the spontaneous action-potentials turned out to be atrial-like. However, as early as on day 11 after transduction, spontaneous contractions were observed and many colonies beat forcefully by day 15. This very rapid induction of beating, compared to the direct reprogramming approaches, led the authors to speculate that pluripotency reprogramming factors (especially *Oct4*) initially “erase” cell identity by epigenetic mechanisms, and soluble factors in a chemically defined medium are then capable of inducing the desired cell type. A few years later, the same group [31] demonstrated that *Oct4* alone, together with a small-molecule cocktail consisting of SB431542 (ALK4/5/7 inhibitor), CHIR99021 (GSK3 inhibitor), parnate (LSD1/KDM1 inhibitor), and forskolin (adenylyl cyclase activator) (SCPF), was sufficient to “erase” the original cell identity, thereby enabling a cell conversion with lineage-specific soluble signals. In this case, BMP4 was added from day six after transduction to induce a cardiomyocyte phenotype. By using this strategy they observed contracting clusters from day 20 and generated 99 ± 17 beating foci on day 30 after 10,000 MEFs were initially plated. Action potential measurements and immunostaining against *myosin light chain 2v* revealed that most of the induced cardiomyocytes are of the ventricular subtype, whereas very few cells displayed atrial or nodal features. Since ventricular cardiomyocytes are typically lost in heart failure, this condition is highly desirable for direct cardiac reprogramming [31]. Additionally, smooth muscle cells as well as endothelial cells could be generated by switching to appropriate cell-differentiation conditions after the treatment with *Oct4* and SCPF, suggesting the generation of nascent cardiac precursor cells during a critical reprogramming window [31].

Likewise, using similar approaches, neural (e.g., [41,42,43]) as well as endothelial [44] and definitive endodermal [45] cells have also been generated from murine and human fibroblasts.

### 2.4. Path of Conversion—Do Cells Pass through a Pluripotent or Progenitor State?

Several groups have investigated the path of direct cell conversion to exclude that those cells pass through a kind of pluripotent or precursor stage before finally adopting their desired cell fate. Interestingly, direct reprogramming approaches with transcription factors do not seem to induce progenitor states, monitored by early cardiac markers like *Mesp1*, *Flk1*, *Isl1*, *Gata4*, and *Nkx2.5*. Rather, it seems to indeed be a direct conversion of fibroblasts into induced cardiomyocytes [9,28,34]. Muraoka *et al.* [9] used FACS sorted MEFs originating from *Mesp1*-Cre x Stop-GFP reporter mice (GFP^−^/Thy1^+^) and transduced them with GMT + miR-133. In this transgenic model early *Mesp1* positive cardiac precursors are permanently labeled by GFP and their fate can be further followed (lineage tracing). Since no GFP expressing *TropT* positive cells were observed after reprogramming it was concluded that iCMs were generated without passing through a mitotic *Mesp1*-positive mesodermal progenitor cell stage. Moreover, they followed *Isl1* gene expression during the initial seven days post transduction and did not observe an upregulation compared to untransduced controls, excluding the involvement of second heart field progenitor cells. Nam *et al.* [28] performed lineage tracing experiments with fibroblasts obtained from an *Nkx2.5*-Cre x R26^tdTomato^ mouse line. Although they found some rare tdTomato-red positive cells after transduction with GMT + *Hand2*, none of these cells became positive for a cardiomyocyte marker, indicating no contribution of first heart field progenitors to induced cardiomyocytes.

In contrast, using exclusively miRs for direct reprogramming, fibroblasts seemed to pass through a rapid progenitor cell state before converting into iCMs [24]. One to five days after transfection with the miR-combination 1, 133, 208, and 499, Jayawardena *et al.* showed an mRNA-upregulation of *Mesp2*, which is considered an early cardiac mesodermal marker. However, no induction of pluripotency markers like *Oct4* or *Nanog* was seen, so a passage through a pluripotent cell type could be excluded. Similarly, other investigators [34] checked for a pluripotent state but did not find any evidence for a transient pluripotent induction prior to conversion into iCMs (no RNA expression of *OCT4*, *SOX2*, *NANOG*, and *TDGF1*).

However, the cell-activation and signaling-directed (CASD) approaches using pluripotency factors combined with a signaling-directed lineage conversion by cytokines or small molecules, guide their cells through a precursor state before becoming iCMs [30,31]. A robust expression of typical cardiac progenitor cell markers like *Nkx2.5*, *Gata4*, *Isl1*, or *Flk1* starting on day nine or 10 after transduction was observed. Furthermore, *Mesp1* expression (as a marker of early cardiac mesoderm), starting on day four and peaking on day 10, was monitored. It was shown that *Isl1*-positive cells were mitotically active precursor cells (Ki67 immunostaining) and may be similar to multipotent *Isl1*-positive cardiovascular progenitors [46], offering the opportunity to expand them in culture and differentiating them in other cardiovascular lineages as well [30,31]. These results were confirmed by Wang and colleagues [31], who demonstrated, using an *Isl1*-Cre lineage tracing model, that *Isl1*-positive cells were generated after the addition of BMP4 and afterwards spontaneously converted into beating clusters. However, it was postulated that during the CASD reprogramming process no pluripotent intermediate was generated that would contribute to iCM induction [30,31]. Using murine embryonic fibroblasts generated from an *Oct4*-promotor-GFP mouse line and monitoring the CASD reprogrammed cells over a 25-day period, no GFP-positive cells could be observed. Other pluripotent marker gene expression, like *Nanog*- or *Rex1*-expression, was nearly undetectable, further supporting the fact that the endogenous pluripotency circuitry remains silenced. Recent research contradicts this finding, though. Maza *et al.* [47] clearly showed that murine fibroblasts reprogrammed with the CASD method according to the abovementioned protocols [30,41], regardless of directing them into a cardiac or neuronal direction, went through a transient state of induced pluripotency before adopting their determined fate. By reprogramming embryonic fibroblasts of a *Nanog*-Cre-tdTomato lineage tracing mouse (*Nanog* indicates pluripotency) into induced neuronal cells (iNCs) or iCMs, approximately 82% of *Sox1* positive iNCs and 93% of *myosin* positive iCMs were tdTomato red positive, indicating a transient acquisition of pluripotency. A second independent study reached similar conclusions by using a different lineage tracing system. Bar-Nur *et al.* [48] demonstrated that virtually all iNCs reprogrammed from fibroblasts of an *Oct4*-Cre-YFP mouse originated from cells that transiently expressed *Oct4*, another marker of pluripotency. An ablation of *Oct4* positive cells by diphtheria toxin A completely prevented iNC formation. The authors therefore suggested that reprogramming by the CASD method requires a passage through a transient pluripotent state.

### 2.5. The Problem with Direct Reprogramming of Human Cells

Interestingly, it is not possible to achieve similar transdifferentiation efficiencies in human cells (independent of the kind of fibroblasts) by using identical combinations of transcription factors, microRNAs, and small molecules found to convert murine cells. The same effect could be observed for neuronal cells. Three factors (*Brn2*, *Ascl1*, and *Mytl1*) were sufficient to reprogram murine cells into functional neurons but they failed to induce a similar program in human fetal fibroblasts. An additional factor (*NeuroD1*) was necessary to convert human fibroblasts into functional neurons [49].

However, reprogramming from somatic cells into induced pluripotent stem cells (iPSCs) works with a similar efficiency and the same four factors, *Oct3/4*, *Sox2*, *Klf4*, and *c-Myc*, for mouse as well as for human cells. Culture conditions after the viral transduction are slightly different, though; in mouse cells leukemia inhibitory factor (LIF) is used for maintenance of pluripotency while human iPSCs need fibroblast growth factor (bFGF).

Nam *et al.* [32] were the first to report a direct conversion of human fibroblasts into iCMs. They showed that a combination of *GATA4*, *TBX5*, *HAND2*, *MYOCD*, miR-1, and miR-133 was sufficient for cardiac reprogramming and achieved 20% of TROPT expressing cells in a starting pool of neonatal human foreskin fibroblasts (HFFs) (see also Table 2). With adult cardiac (aHCFs) or dermal fibroblasts (aHDFs) they only achieved 13% TROPT expressing cells or 9.5%, respectively. However, only rare beating cells could be observed after a long period in culture (after 11 weeks, only from HCFs). Fu *et al.* [34] could induce calcium transients after four weeks in 20% of starting cells (human H9 embryonic stem cells derived fibroblasts carrying an *αMHC*-mCherry reporter) by overexpressing GMT together with *MESP1*, *MYOCD*, and a nuclear hormone receptor called *ESRRG*, but failed to induce human iCMs by GMT alone. Beating events were not reported. Like Fu *et al.* [34], another group [33] using a similar combination of transcription factors were able to reprogram human cardiac and dermal fibroblasts (HCFs, HFFs) into cells with properties of human cardiomyocytes. By transduction with GMT + *MESP1* and *MYOCD* they generated about 5% αACTININ and TROPT positive cells from HCFs (patients were between 3–5 months) and about 1% cells with Ca^2+^ oscillations after four weeks of culture. However, even after a prolonged time of culture they did not observe spontaneous beating (only in co-culture with neonatal rat cardiomyocytes). Recently, Muraoka *et al.* [9] induced between 2% and 8% of αACTININ/TROPT double positive cells by a lentiviral transduction of GMT + *MESP1* and *MYOCD* into HCFs. Interestingly, they were able to increase the percentage of iCMs to 23%–27% by adding miR-133 to the reprogramming cocktail.

Based on the abovementioned, as opposed to the straight-forward generation of iPSCs, all published results so far implicate that the conversion of fibroblasts into cells with a cardiomyocyte-like phenotype is not easily transferable from murine models to the human system. Therefore, further research is essential to identify optimal reprogramming tools (transcription factors, miRNAs, *etc.*) as well as culture conditions (small molecules, *etc.*) for improving reprogramming efficiency and quality of iCMs.

### 2.6. Reliable Markers and Transdifferentiation of Induced Cardiomyocytes (iCMs)

The fact that different groups are using various markers and reporter systems to define the maturity of iCMs in their approaches makes results hardly comparable among each other.

**Figure 2:**
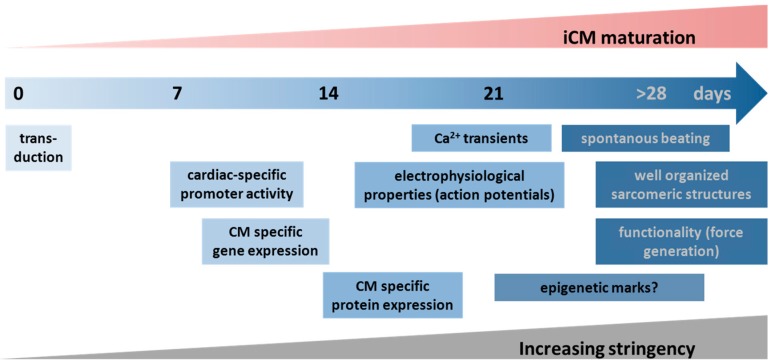
Reliability and temporal appearance of cardiomyocyte specific markers during the direct reprogramming process. Different markers for induced cardiomyocytes (iCMs) are depicted with increasing stringency to determine the cardioinducing effect and cellular reprogramming capacity of certain factor combinations.

In the first published reports (e.g., [15,22,50]) the evaluation of reprogramming efficiency mostly relied on non-functional measurements, such as the detection of fluorescent proteins driven by the activation of cardiomyocyte-specific promoters or the expression of cardiac-specific genes including those encoding for cardiac cytoskeletal proteins, cardiac transcription factors, or cardiomyocyte ion channel proteins [25,26]. These markers combine two advantages: (1) they are readily detectable by flow cytometry or real-time PCR and (2) they can be detected quite early (after 1–2 weeks) post transduction (see also Figure 2). Therefore, these markers are especially valuable for large screening approaches. However, a more reliable approach asserting a complete conversion of fibroblasts into cardiomyocytes would be a proof of cytoskeletal reorganization and the detection of well-organized and cross-striated myofibrils [26]. Nam *et al.* [28] showed that a well-organized sarcomere structure is the *conditio sine qua non* to facilitate spontaneous beating in transduced iCMs. Since sarcomeric protein expression is not strictly correlated with sarcomeric protein organization, most of the current estimates of reprogramming efficiency solely based on sarcomere protein expression may be vastly overestimated. Therefore, immunostaining for sarcomeric proteins and, more importantly, the evaluation of their organization grade should be assessed after iCM induction. Finally, the most stringent marker for showing successful conversion of fibroblasts into iCMs is the evidence of spontaneously contracting myocytes. This marker is unfortunately also not readily quantifiable in large numbers of cells screened in reprogramming experiments [25]. To this end, Addis *et al.* [25] described a method focusing on the detection of rhythmic intracellular calcium level oscillations, which is regarded as the electrophysiological substrate of cardiomyocyte contraction. In order to visualize and quantify myocyte contraction, they constructed a reporter system in which the calcium indicator GCaMP (coupled with a GFP fluorescence) is driven by the cardiomyocyte-specific *TroponinT* promoter. This seems to be a more stringent outcome compared to fluorescent proteins driven by cardiomyocyte-specific promoters [27].

The use of ambiguous transgenic reporter models or impure starting cell populations may lead to false-positive results and are prone to misinterpretations. For example, unspecific transgene activation may result in an overestimation of the conversion efficiency. The impurity of applied starting cell populations is another subject of controversial discussion. Using murine embryonic fibroblasts (MEFs), cardiac progenitor cells could occur in this starting cell population or a kind of spontaneous differentiation of immature cells could lead to the emergence of putative iCMs. Similarly, if cardiac fibroblasts are utilized as a starting cell population they could be also “polluted” by cardiac progenitor cells or even cardiomyocytes.

## 3. Direct Lineage Reprogramming of Fibroblasts into Cardiomyocytes *in Vivo*

Interestingly, *in vivo* approaches of direct cardiac reprogramming applied after experimental myocardial infarction in mice revealed better efficiencies than *in vitro* approaches (see also Table 3). Qian *et al.* [50] and Song *et al.* [22] both used classic genetic lineage tracing models to proof that direct cardiac reprogramming transdifferentiates non-myocytes into functional iCMs in the injured murine heart. Qian *et al.* [50] directly injected retroviruses coding for GMT into infarcted myocardium of *Periostin*-Cre x R26^LacZ^ and *Fsp1*-Cre x R26^LacZ^ mice (*Periostin* and *Fsp1* are fibroblast markers [51]). Retroviruses only infect proliferating cells. Therefore, cardiac fibroblasts in the infarction border zone would be targeted but resting cardiomyocytes would not. Approximately 35% of cardiomyocytes in the border/infarct zone were considered newly generated iCMs derived from resident cardiac fibroblasts. Furthermore, functional studies three months after myocardial infarction revealed that retroviral GMT gene transfer significantly improved cardiac function and reduced the degree of fibrosis. Song and colleagues [22] reported that GMT + *Hand2* retroviral injection into murine ischemic hearts converted endogenous cardiac fibroblasts into functional iCMs *in situ* (between 2% and 6%). For lineage tracing they used an *Fsp1*-Cre x R26^LacZ^ mouse and an inducible *Tcf21*-iCre x R26^tdTomato^ mouse (*Tcf21* is also a fibroblast marker [51]) to prove that iCMs were derived from cardiac fibroblasts. Three months after experimental left coronary artery (LCD) ligation, the group could demonstrate that the ejection fraction increased twofold and that the scar size was reduced by 50% in GMT + *Hand2* treated mice compared to untreated controls. By performing pulse-labeling experiments with tamoxifen-inducible αMHC-iCre x R26^Reporter^ mice and labeling endogenous cardiomyocytes, both groups ruled out that newly generated iCMs were induced by cell-fusion events [22,50]. Likewise, Inagawa *et al.* [36] retrovirally co-transduced GMT and reporter genes (GFP or DsRed) into the infarcted myocardium of immunosuppressed mice to further promote the survival of the virally transduced cells. They proceeded on the assumption that virally infected cells may be rejected by an intact immune system. With this approach, they demonstrated that about 1% of cells were newly generated iCMs (αActinin as CM marker). Notably, they did not observe significantly more iCMs but more mature induced cardiomyocytes when using a polycistronic GMT vector. Thus, they postulated that polycistronic vectors may be a valuable tool for *in vivo* cellular reprogramming strategies.

Mathison *et al.* [52] applied GMT to infarcted rat hearts three weeks after LCD ligation and also achieved quite an improvement in heart function seven weeks after myocardial injury. The extent of fibrosis was reduced by half and the ejection fraction was significantly improved after administration of GMT compared to controls. The authors could even enhance the benefit by adenovirally applying VEGF together with GMT.

Jayawardena *et al.* used a lentiviral approach, delivering a microRNA cocktail (miR-1, -133, -208, and -499) into the infarcted hearts of *Fsp1*-Cre x R26^tdT^°^mat^° mice to trace the conversion of fibroblasts into iCMs [24]. As opposed to retroviruses, lentiviruses do not only infect proliferating cells but also cardiomyocytes and other cardiac non-dividing cells. Quantification of cells positive for tdTomato red (indication of a fibroblast cell) and TropT revealed that the infarct region contained up to 1% of newly generated iCMs from resident fibroblasts. Parameters of heart function, however, were not evaluated in these experiments. Recently, the same group [53] performed functional studies to clarify the open questions and demonstrate improvement of cardiac function by lentiviral delivery of miR-1, miR-133, miR-208, and miR-499.

The remarkably higher efficiency of *in vivo* reprogramming may be explained by the cardiac microenvironment providing soluble co-factors, an exposure to the extracellular matrix, or an influence of mechanical forces during reprogramming, thereby enabling a more rapid conversion of resident fibroblasts into cardiomyocytes [1,2,5]. Furthermore, presumably promoted by the same mechanisms, the *in vivo*-generated iCMs were more mature than their *in vitro* counterparts and showed morphology and function much more similar to those of endogenous cardiomyocytes [1]. Careful studies with different genetic lineage tracing models demonstrated that the newly generated iCMs most likely arose from a true *in vivo* conversion of resident non-myocytes rather than leaky Cre expression or cell–cell fusion [2].

**Table 3:** Direct cardiac reprogramming *in vivo* (rodent infarction by LCD ligation).

Reference	Genetic Mouse Model	Application Form	Reprogramming Factors	iCMs Percentages/When	Functional Improvements
[50]	Periostin-Cre x R26^LacZ^ Fsp1-Cre x R26^LacZ^ αMHC-MerCreMer x R26^eYFP^	Retroviral delivery	GMT	Periostin-Cre x R26^LacZ^ (4 weeks): ~35% iCMs (β-Gal^+^ and αActinin^+^)	Yes (MRI, serial echo), blinded study, 3 months after MI
[22]	Fsp1-Cre x R26^LacZ^ Tcf21-iCre x R26*^td^*^Tomato^ αMHC-MerCreMer x R26^LacZ^	Retroviral delivery	GMTH_2_	Fsp1-Cre x R26^LacZ^ (4 weeks): ~6.5% iCMs (β-Gal^+^) Tcf21-iCre x R26*^td^*^Tomato^ (3–4 weeks): ~2.4% iCMs (tdTomato^+^ after Langendorf perfusion method)	Yes (MRI, echo), blinded study, 3 months after MI
[36]	Immunosuppressed mice (nude mice), no lineage tracing	Retroviral delivery	GMT; GMT polycistronic	GFP: no αActinin in GFP+ cells (2 weeks) GMT + GFP: ~1% of GFP+ cells express αActinin (2 weeks) GMT polycistronic + GFP: ~0.8% of GFP+ cells express αActinin (2 weeks)	N.A.
[24]	Fsp1-Cre x R26*^td^*^Tomato^ x αMHC-CFP	Lentiviral delivery	miR-1, -133, -208, -499	~1% iCMs (CFP^+^ and tdTomato^+^) (6 weeks)	N.A.
[52]	Fisher 344 rats	Adenoviral (VEGF) & lentiviral (GMT) delivery	VEGF (directly after MI), GMT (3 weeks after MI)	N.A.	Yes (serial echo), 7 weeks after MI (best: GMT + VEGF)
[53]	Fsp1-Cre x R26*^td^*^Tomato^	Lentiviral delivery	miR-1, -133, -208, -499	~12% iCMs (tdTomato^+^ and TropT^+^) (7 weeks) (*cave: NegmiR: 4%*)	Yes (serial echo), 3 months after MI

Abbreviations: LCD: left coronary artery; iCMs: induced cardiomyocytes; N.A., not available; Genetic Mouse Models: R26: ROSA26 Locus; iCre: inducible Cre; Reprogramming factors: G: Gata4; M: Mef2c; T: Tbx5; H_2_: Hand2; miR: microRNA; VEGF: vascular endothelial growth factor; MI: myocardial infarction; Functional Improvements: echo: echocardiography; MRI: magnetic resonance imaging.

Although higher conversion rates have been observed *in vivo* than *in vitro*, Yi *et al.* [3] believe that the numbers of new cardiomyocytes are still too low to be a likely explanation for all the improvements detected in cardiac function. It is conceivable that the amount and paracrine behavior of scar-producing fibroblasts is changed by virus application, which may further contribute to reduced scar sizes after experimental infarctions [1]. Paracrine signals derived from reprogrammed fibroblasts may improve the performance of pre-existing cells and, thus, cardiac function [1]. Similar mechanisms have been demonstrated to play a role after myocardial cell transplantation [1,4]. Other facts that may contribute to the increased efficiency *in vivo* could be attributed to a self-concentration of the virus within the relatively small extracellular compartment in the adult heart associated with a greater biological effect locally [5].

## 4. Underlying Mechanisms

The underlying molecular mechanisms of cell conversion during direct reprogramming remain poorly understood. However, as with iPSCs, the suppression of the starting-cell epigenetic signature is the key to overcoming one major molecular roadblock for successful reprogramming, namely the shutdown of the fibroblast program, before an adoption of the desired cell fate (iCM) becomes possible.

Muraoka *et al.* [9] proposed that cardiac reprogramming from fibroblasts into iCMs by using GMT + miR-133 is mediated by a direct repression of the transcription factor *Snai1*, which is a master regulator of epithelial-to-mesenchymal transition and a direct target of miR-133. *Snai1* is able to induce mesenchymal programs and fibrogenesis during development and disease [9]. By knocking down *Snai1*-expression with siRNA in GMT-transduced MEFs, Muraoka and colleagues could significantly increase the reprogramming efficiency compared to GMT alone. They observed an upregulation of a panel of cardiac genes, spontaneous Ca^2+^ oscillations, and cell contractions, although the level seen with GMT + miR-133 was not reached. By overexpressing *Snai1* during the reprogramming process, cardiac gene expression and spontaneous beating were inhibited. Similar results were obtained for human cardiac fibroblasts, suggesting an important role for *Snai1* during the reprogramming process of fibroblasts into cardiomyocytes in mice and men.

Ifkovits *et al.* [27] postulated that an inhibition of TGFβ (Transforming Growth Factor β) may play an important role in early reprogramming events during the conversion of murine embryonic fibroblasts into iCMs. They could show a five-fold improvement of iCM induction when a TGFβ-inhibitor (SB431542) was used in addition to a combination of GMT + *Hand 2* + *Nkx2.5*. Further, they excluded that this improvement was induced by proliferation of iCMs or apoptosis of non-reprogrammed MEFs. Since TGFβ can act as an activator of *Snai1* [9], the theories complement each other. Fu *et al.* [34] also found that TGFβ-signaling was important for the efficiency of direct cardiac reprogramming in human cells. Conversely, they observed a positive effect of adding TGFβ1 to their five-factor TF cocktail (GMT+ *MESP1* + *ESRRG*) and could reverse this effect with SIS3, which specifically inhibits *SMAD3*, a transcription factor that is activated downstream of TGFβ-signaling.

## 5. Remaining Challenges of Direct Reprogramming

**Figure 3:**
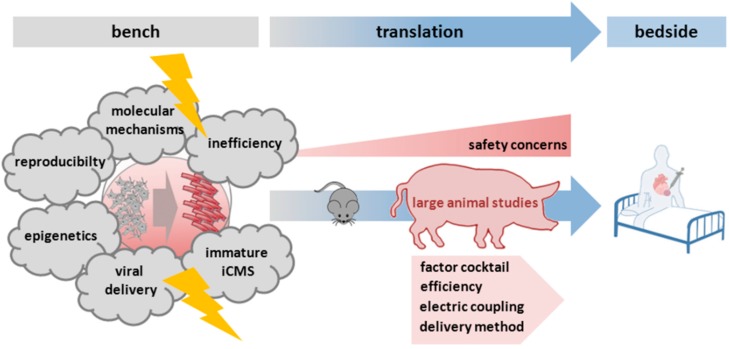
Remaining challenges of direct reprogramming approaches before a clinical application. Before a translation of direct reprogramming approaches from bench to bedside, several challenges have to be resolved. First of all, issues like the inefficiency of the reprogramming process from fibroblasts (grey cells in front of the arrow) to induced cardiomyocytes (iCMs, red elongated shaped cells behind the arrow), the insufficient maturity of the iCMs, the delivery method, or the understanding of underlying mechanisms have to be addressed. As a next step, especially for evaluating the safety of delivery methods, large animal studies have to be performed. Further effort has to be put into the optimization of the reprogramming technology before a clinical application becomes possible.

Despite initial enthusiasm regarding the enormous potential of a direct cardiac lineage conversion for future regenerative therapies, there are still considerable challenges that are far from being resolved. First of all, a direct cardiac reprogramming approach implies the complete adoption of all morphological and functional features of the destination cell type, in this case a cardiomyocyte. Furthermore, a resetting of the epigenetic features of the starting cell type is required, as well as the downregulation of the transcriptional activity of transduced transgenes [20] (see also Figure 3).

### 5.1. Inefficiency of the Reprogramming Process

The induction efficiency of fully reprogrammed functional cardiomyocytes *in vitro* remains low. This is not surprising considering the induced pluripotent stem cell reprogramming efficiency rate of 0.01%–0.1%, which is likely because of major epigenetic barriers that cells cannot easily overcome [2]. The conversion rate *in vivo* (1% to 35%), although higher than *in vitro*, is not sufficient for a full regeneration of injured myocardium. The conversion efficiency from fibroblasts into neurons, for example, has been reported to range from 0.4% to 5.9% *in vivo* [54]. Interestingly, this and other groups see no difference between the conversion efficiency from fibroblasts into neurons *in vivo* and *in vitro* [49,55,56].

Why is the efficiency so important? Since directly reprogrammed fibroblasts exit the cell cycle very rapidly and thus are no longer proliferative, efficiency is of much greater concern than for iPSCs, which adopt a proliferative cell fate [2]. The initial reprogramming rate has to be high enough to be considered for therapeutic uses [5]. Besides the efficiency, speed and quality of conversion from fibroblasts to cardiomyocytes should also be sufficiently high. Chen *et al.* [5] predicted that at least 50% of the starting cell population should be converted into fully mature cardiomyocytes within five to 10 days post transduction for this strategy to be considered therapeutically relevant in a post-infarct setting.

### 5.2. Viral Delivery

A major obstacle for future clinical translation is that current reprogramming approaches are mostly applying viral delivery methods. These methods carry the risk of random genetic integration of virally overexpressed transgenes and moreover the risk of tumor formation [1,5].

Although the addition of small molecules [31] can reduce the number of virally delivered reprogramming factors (mostly CASD approaches) up to now these approaches have not worked without at least one virally-delivered transcription factor. Despite a number of current clinical trials addressing viral gene therapy and a recent approval of a gene therapy drug for lipoprotein lipase deficiency, the safety of viral vectors remains a concern [2,57].

For future translation into clinical applications, other forms of factor delivery like non-integrating vectors (adenovirus-associated vectors) or a replacement of reprogramming factors by small molecules are inevitable [1,2]. However, it remains unclear whether small molecules will be sufficient for *in vivo* reprogramming since they do not specifically target one cell type like fibroblasts but may also influence remaining healthy cardiomyocytes. Another alternative approach for *in vivo* reprogramming obviating viral delivery methods could be the application of so-called modified mRNAs that were codon-optimized and chemically modified to increase stability against the innate immune system [58].

### 5.3. Molecular Mechanisms Insufficiently Defined

Despite ongoing progress in the understanding of the mechanistic basis, the precise molecular mechanisms of the cardiac reprogramming process are still rather unknown, even in mice [30]. As mentioned earlier, it is not unlikely that there might be a mechanistic difference on one or more levels between the reprogramming processes in murine and human cells [59]. The differences may only lie in the selection of indispensable transcription factors or may go down to the epigenetic level. The key role thereby may be the induction of a developmentally more naive, open-chromatin state marked by a higher epigenetic instability than the starting cell type [30]. Especially for an improvement of efficiency, a more detailed understanding of the molecular underpinnings of direct reprogramming and its various intermediate stages is required.

### 5.4. Epigenetics

Many groups have analyzed the epigenetic changes in reprogrammed cell populations. However, only few loci can be checked by standard approaches with chromatin immunoprecipitation assays and subsequent surveying of trimethylation-levels of histone H3 Lys4 (H3K4me3) and Lys27 (H3K27me3). An enrichment of H3K4me3 marks transcriptionally active chromatin, whereas H3K27me3 marks transcriptionally inactive chromatin [34]. By this approach, several groups demonstrated an enrichment of H3K4me3 in selected cardiac-specific genes in murine and human settings and an enrichment of H3K27me3 in fibroblast- or pluripotency-specific promoters in the induced cardiomyocyte cell population [30,34]. This suggests that fibroblast-specific gene programs were downregulated, thereby facilitating the activation of cardiomyocyte-specific genes.

Another tool to check the DNA methylation is a method called bisulfite genomic sequencing. In general, hypermethylated promoters block gene transcription while demethylated promoters allow transcriptional activity. Fu *et al.* [34] analyzed the methylation state of selected promoter regions (*MYH6* (=*αMHC*), *MYH7*, and *MYL7*) in a starting population of differentiated human fibroblasts, compared to iCMs at two and four weeks post transduction as well as differentiated human CMs. As expected, the three mentioned promoter regions were hypermethylated in fibroblasts but relatively demethylated in iCMs, similar to differentiated human CMs. Certainly, it would be more desirable to compare iCMs to *in situ* cardiomyocytes. A comparison of the methylation state between the starting cell population, the reprogrammed cells, and the desired cell population by bisulfite genomic sequencing would be required to get full information about the completeness of cell type conversion, at least from an epigenetic point of view. Furthermore, it has to be evaluated whether the converted cell type is epigenetically stable over a longer time period post induction.

It may be an issue, especially in CASD approaches, that epigenetically unstable intermediate cell populations could be generated during the reprogramming process with pluripotency factors, which could give rise to a multitude of cell types as they rapidly “relax” back into epigenetically more stable states [30].

### 5.5. Induced Cardiomyocytes—An Immature and Heterogeneous Cell Population?

Another important point of concern is the fact that directly reprogrammed induced cardiomyocytes are relatively immature—morphologically as well as functionally [59]. As already mentioned above, cardiomyocytes are a very complex cell type with elaborate sarcomeric structures and advanced electrophysiological tasks that usually do not proliferate in their mature form. It is much more ambitious to generate such a complex cell type than to generate undifferentiated pluripotent stem cells.

Furthermore, iCMs are not homogeneous in their maturation state but exhibit rather heterogeneous features, e.g., concerning their levels of sarcomere organization [28]. Interestingly, the maturity level of iCMs seems to be higher *in vivo*. Regarding the latter, some studies suggested that the presence of mechanical force, extracellular matrix, and secreted proteins might improve the reprogramming efficiency and maturation of iCMs [59]. How long does it take to get a mature cardiomyocyte by *in vitro* culture? A recent publication [60] compared the maturation state of 20-day and one-year-old *in vitro* differentiated cardiomyocytes (from a human iPSC line). One-year-differentiated cardiomyocytes were more similar to human *in vivo*-derived adult heart tissue than to human three-month-old cardiac samples, indicating an almost mature state after one year of *in vitro* culture. Pathways related to hypertrophic signaling, sarcomere organization, calcium, and cAMP-mediated signaling and integrin signaling were significantly upregulated in one-year-differentiated cardiomyocytes and adult heart tissue. However, 20-day-differentiated cardiomyocytes were strongly different from adult heart samples and even from the three-month-old cardiac tissues. An array analysis revealed that the let-7 microRNA family is an important mediator during the maturation of cardiomyocytes, especially for the regulation of metabolic energetics.

For the future and especially for the translation to a clinical application of reprogramming approaches, important safety issues may arise from only partially reprogrammed cells in the heart potentially resulting in disturbances of cardiac rhythm because of insufficient electrical coupling [57]. Although Qian and colleagues [50] did not detect any arrhythmias in the hearts of mice that had received the reprogramming factors, it will be necessary to further evaluate this in large animal models [57].

### 5.6. Reproducibility in Different Labs—Methodological Issues

Since the outcome of direct reprogramming obviously differs in various labs all over the world, and not only on the basis of evaluating efficiency by different markers, researchers suggest that an accumulation of small differences in the work flow, like culture conditions and isolation methods for the starting fibroblast population, the fibroblast line, or the method of virus production, might cause reproducibility issues [59]. It is recommended that fresh, non-senescent fibroblasts (low passage numbers) and high virus titers (expressing the reprogramming factors) should be used for achieving successful cardiac reprogramming [1]. Furthermore, stringent and uniform standards for the characterization of iCMs are required for helping to guide the generation of fully functional cells [5]. For the future advancement of this direct reprogramming techniques and an easier transfer between different labs, it will be necessary to work on the standardization of the protocols [1].

## 6. Summary and Future Perspective

Since the landmark discovery of the method to transdifferentiate somatic cells into iPS cells by overexpressing a set of defined transcription factors in 2006 [6], researchers from all over the world soldier on identifying the best strategies for directly reprogramming fibroblasts into functional cardiomyocytes without passing through a pluripotent state. Several strategies using different reprogramming factors, microRNAs, small molecules, and defined medium conditions have been tested with variable and minor success *in vitro*. Interestingly, *in vivo* approaches in murine models of myocardial infarction have been much more promising and blinded animal studies could demonstrate significant functional improvements after myocardial ischemia. Nevertheless, many questions and challenges remain to be resolved before this technology can be translated safely into clinical applications. Unfortunately, until now, no robust method or combination of transcription factors, miRNAs, or small molecules has been found to efficiently transdifferentiate/reprogram different types of fibroblasts of murine and human origin into mature, fully functional cardiomyocytes.

Overall, this new technology possesses great potential for a “true” cardiac regeneration therapy. In the future a patient with a recent myocardial infarction could undergo a treatment regimen whereby a reprogramming cocktail is introduced directly into the tissue at the infarction site for reprogramming endogenous scar fibroblasts into mature and functional cardiomyocytes that restore cardiac function by increasing the cardiomyocyte content and diminishing the scar size [5]. In this setting, a different target cell population would be imaginable: monocytes that infiltrate the site of injury. Recently, it was postulated that two different subsets of monocytes play a role during the first two weeks after myocardial infarction in a biphasic manner. During the first four days, proinflammatory Ly-6C_high_ monocytes promote digestion of infarcted tissue and removal of necrotic debris whereas after that reparative Ly-6C_low_ monocytes propagate repair [61]. Monocytes descend from macrophage and dendritic cell progenitors from the bone marrow and can give rise to either of the mentioned mature cell types depending on the environment. This suggests a remarkable “plasticity” and therefore predestinates this cell population as a reprogramming target.

This kind of *in vivo* reprogramming approach has several advantages over cell transplantation therapies. Since reprogramming factors are directly injected into the heart, no issues arise concerning the homing, survival, or migration of the transplanted cells [1]. The risk of tumor formation is lowered because induction of pluripotency is avoided before cardiac differentiation. In addition, as most heart diseases are associated with an increase in cardiac fibrosis, this method might be applied to nearly any type of heart disease [1]. However, before a clinical application of this technology could become reality, the postulated beneficial effects of direct cardiac conversion and the safety of the delivery methods must be demonstrated in large animals [1,59]. Further effort has to be put into the optimization of reprogramming human cells [57]. In the future it is possible that *in vivo* direct reprogramming therapies to regenerate damaged tissue will become the new paradigm for many human diseases [2].
